# Expression of SATB1 and HER2 in breast cancer and the correlations with clinicopathologic characteristics

**DOI:** 10.1186/s13000-015-0282-4

**Published:** 2015-05-09

**Authors:** Xiangdong Liu, Yan Zheng, Chuanwu Qiao, Fei Qv, Jingnan Wang, Butong Ding, Yuping Sun, Yunshan Wang

**Affiliations:** Key Laboratory for Reproductive Medicine of Shandong Province, Provincial Hospital Affiliated to Shandong University, Jinan, China; Central Laboratory, Jinan Central Hospital Affiliated to Shandong University, Jinan, China; Department of Pharmacy, Jinan Central Hospital Affiliated to Shandong University, Jinan, China; Department of Pathology, Jinan Central Hospital Affiliated to Shandong University, Jinan, China; Medical Research & Laboratory Diagnostic Center, Jinan Central Hospital Affiliated to Shandong University, Jinan, China; Department of Oncology, Jinan Central Hospital Affiliated to Shandong University, Jinan, China; Shandong Province Key Laboratory for Target Molecule, Jinan, China; Current Affiliation: Department of Thoracic Surgery, Cancer Insititute and Hospital, Chinese Academy of Medical Sciences and Peking Union Medical College, Beijing, China

**Keywords:** Breast cancer, SATB1, HER2, HR, Tumor histological grade, Prognosis

## Abstract

**Background:**

Special AT-rich sequence binding protein 1 (SATB1) is found acting as a “genome organizer” that functions as a landing platform to regulate tissue-specific gene ex-pression. In breast cancer cell lines it has been proven that SATB1 could upregulate the expression of the HER2. In this paper, the relevance of SATB1 and HER2 expression was assessed in human breast cancer tissues, and their influence on tumor histological grade and patients’ survival was explored.

**Methods:**

Using immunohistochemistry (IHC) and fluorescence in situ hybridization (FISH), 169 patients with breast cancer were assessed for SATB1 expression, HER2 amplification and hormone-receptor (HR) expression. The effects of SATB1 expression on HER2 and HR expression as well as their association with clinicopathologic characteristics were further analyzed by statistical evaluation.

**Results:**

SATB1 expression was correlated with HER2 expression in breast cancer(*r =* 0.191; *p* = 0.013). SATB1, HER2 and SATB1/HER2 co-expression was negatively correlated with HR expression (*r = −*0.228, *p* = 0.003; *r = −*0.338, *p* = 0.000; *r = −*0.527, *p* = 0.000, respectively). SATB1 and HER2 single positive and their co-expression were all significantly correlated with higher histological grade (*r =* 0.239, *p* = 0.002; *r =* 0.160, *p* = 0.038; *r =* 0.306, *p* = 0.003, respectively). Multivariate cox regression analyses showed that SATB1 and HER2 were independent risk factors for breast cancer patients, while HR was a protective factor for patients’ survival. Comparing to SATB1 or HER2 single positive expression, SATB1/HER2 co-expression tended to have even worse prognosis.

**Conclusions:**

SATB1 and HER2 performed a synergistic effect in breast cancer. Their expression correlated with poorly differentiated breast cancer and indicated an unfavorable prognosis.

**Virtual slides:**

The virtual slide(s) for this article can be found here: http://www.diagnosticpathology.diagnomx.eu/vs/1400555050159723.

## Background

Special AT-rich sequence binding protein 1 (SATB1) has been shown to regulate the expression of more than ten percent of genes by binding to the upstream regulatory regions which directly influence the promoter activity and gene expression [1-3]. SATB1 is reported to carry important weight to the progression of gastric cancer, cutaneous malignant melanoma, breast cancer, lung cancer, and lymphoma [4,5,3,6,7]. A large number of target genes regulated by SATB1 involve in cancer cell proliferation, development and differentiation. Han et al. [3] showed that SATB1 protein was detected in all 16 poorly differentiated infiltrating ductal breast carcinomas, and the human epidermal growth factor receptor 2 (HER2) gene was directly upregulated by SATB1.

HER2, also known as ERBB2, has been recognized as oncogenic protein with tyrosine kinase activity. Phosphorylation of HER2 could trigger downstream signaling events involved in malignant transformation and tumorigenesis, and ultimately result in poor clinical outcomes [8,9].

As a hormone-dependent tumor, ascertainment of estrogen-receptor (ER) and progesterone-receptor (PR) along with HER2 is regarded essential to treatment of breast cancer [10,11]. It is reported that positive expression of hormone-receptor (HR, including ER and/or PR) generally carries a better prognosis, on account of the target of response to endocrine therapy [9,11]. As a predictive marker, HER2 amplification values high in HER2 targeting therapy. In addition, the amplification of HER2 played a negative role in responding to endocrine sensitivity in recent discovery; over-expression of HER2 might lead to resistance to tamoxifen therapy [12]. Thus, the interaction between HER2 and HR is probably directed by complex mechanisms.

In breast cancer progression, Ras/Raf/MEK/MAPK and PI3K/Akt/mTOR signaling pathways are commonly dysregulated by the crosstalk among various growth factors and hormone receptors including HER2 and ER [13]. Several experimental studies demonstrate that the activation of these two signaling pathways by HER2 lead to phosphorylation and activation of ERα [14]. Shigeaki Kato et al. [14] reported that MAPKs could be activated through EGF-HER2 signaling pathway and the activated MAPK was able to phosphorylate the Ser 118 of ERα. PI3K signaling pathway, which is a major signaling hub downstream of HER2 and other receptor tyrosine kinases, could promote anti-estrogen resistance [15]. Herein, HER2 can alter ER’s function and thereby contribute to tumor growth and tamoxifen resistance according to the signaling crosstalk between ER and HER2 pathways. Interestingly, Han et al. [3] reported that MAPK signaling was activated and PI3K/mTOR signaling was suppressed by SATB1 in breast cancer cells. Therefore, the association among SATB1, HER2 and HR in breast cancer is still confounded.

Previous studies have demonstrated that HR expression correlated with low histological grade, while HER2 expression correlated with high histological grade in breast cancer [10,16]. It was reported that the expression of SATB1 was higher in poorly differentiated than in well differentiated breast cancer and completely absent in adjacent normal tissues [3,17]. But to our knowledge, little information has been available on the relationships among the expression of SATB1, HER2 and HR in breast cancer tissues so far.

The objectives of this study were to assess the correlations of SATB1 expression with HER2 and HR expression in breast cancer tissues, and to evaluate the effects of SATB1 expression on HER2 and HR expression as well as their relationships with clinicopathologic characteristics.

## Methods

### Patients and tissue samples

169 breast cancer patients from Jinan Central Hospital Affiliated to Shandong University and Shandong Province Key Laboratory for Tumor Target Molecule (China), treated with surgical resection between January 2000 and December 2010, were studied in this paper. All the individuals were treatment-naive before pathologically identified as breast cancer. Ethical permission was obtained from the Ethics Committee in Jinan Central Hospital. We got all patients’ informed consent prior to their inclusion in our study. The patients were all females and aged 29–81 years (mean 53.6 years). The median follow-up period was 95 months with a range of 2–131 months. Tissue samples were divided into two groups: breast cancer tissues samples (*n =* 169) and corresponding paracancerous normal tissue samples (*n =* 40). Survival data was available in 119 patients. All cancers were histopathologically confirmed as invasive ductal carcinoma (IDC) (*n =* 144), invasive lobular carcinoma (ILC) (*n =* 13) and other types (*n =* 12). Histological grade was classified according to Elston/Nottingham modification of the Bloom-Richardson system [18]. The clinicopathologic variables of the patients were shown in Table 1.

**Table 1:** **Relationships between the expression status of SATB1, HER2 and** **HR and clinicopathologic parameters**

**Parameter**	***n***	**SATB1**			**HER2**			**HR**		
		**+**	**-**	***P***	**+**	**-**	***P***	**+**	**-**	***P***
Age										
≤50	76	40	36	0.672	23	53	0.26	48	28	0.97
>50	93	52	41		21	72		59	34	
Tumor size										
≤2 cm	45	22	23	0.386	10	35	0.499	29	16	0.855
>2 cm	124	70	54		34	90		78	46	
Tumor type										
IDC	144	79	65	0.538	37	107	0.214	90	54	0.879
ILC	13	6	7		5	8		9	4	
Others	12	7	5		2	10		8	4	
Histological grade										
Low	56	21	35	0.002	9	47	0.038	39	17	0.232
High	113	71	42		35	78		68	45	
Lymph node status										
Negative	78	42	36	0.887	19	59	0.648	45	33	0.162
Positive	91	50	41		25	66		62	29	
TNM stage										
I-II	118	61	57	0.279	26	92	0.072	77	41	0.429
III-IV	51	31	20		18	33		30	21	

### SATB1 assessment

All specimens were fixed in neutral 10% buffered formaldehyde, embedded in paraffin, cut into 4-μm-thick slices, and supplied for IHC and FISH test. Immunohistochemistry (IHC) was used to determine SATB1 expression. Rabbit monoclonal anti-SATB1 antibodies bought from Abcam (Code ab92307, Abcam, USA) were used to analyze the expression of SATB1 following the manufacturer’s instructions. Briefly, antigen retrieval was performed before staining; anti-SATB1 antibody was diluted by 1:100 and incubated overnight. After the samples were fully washed, the polymer detection system (PV-9000, ZSGB-BIO, China) was used for IHC staining. Sections were incubated with the secondary polyperoxidase-anti-rabbit IgG antibody, and chromogen substrate reagent DAB were used to detect the antigen. A brown color reaction with distinct morphology was developed with DAB in the peroxidase system. Positive controls were processed using positive specimens which were recommended by manufacturer’s instructions, and negative controls were executed in parallel slices to which the primary antibody were omitted [19].

**Figure 1:**
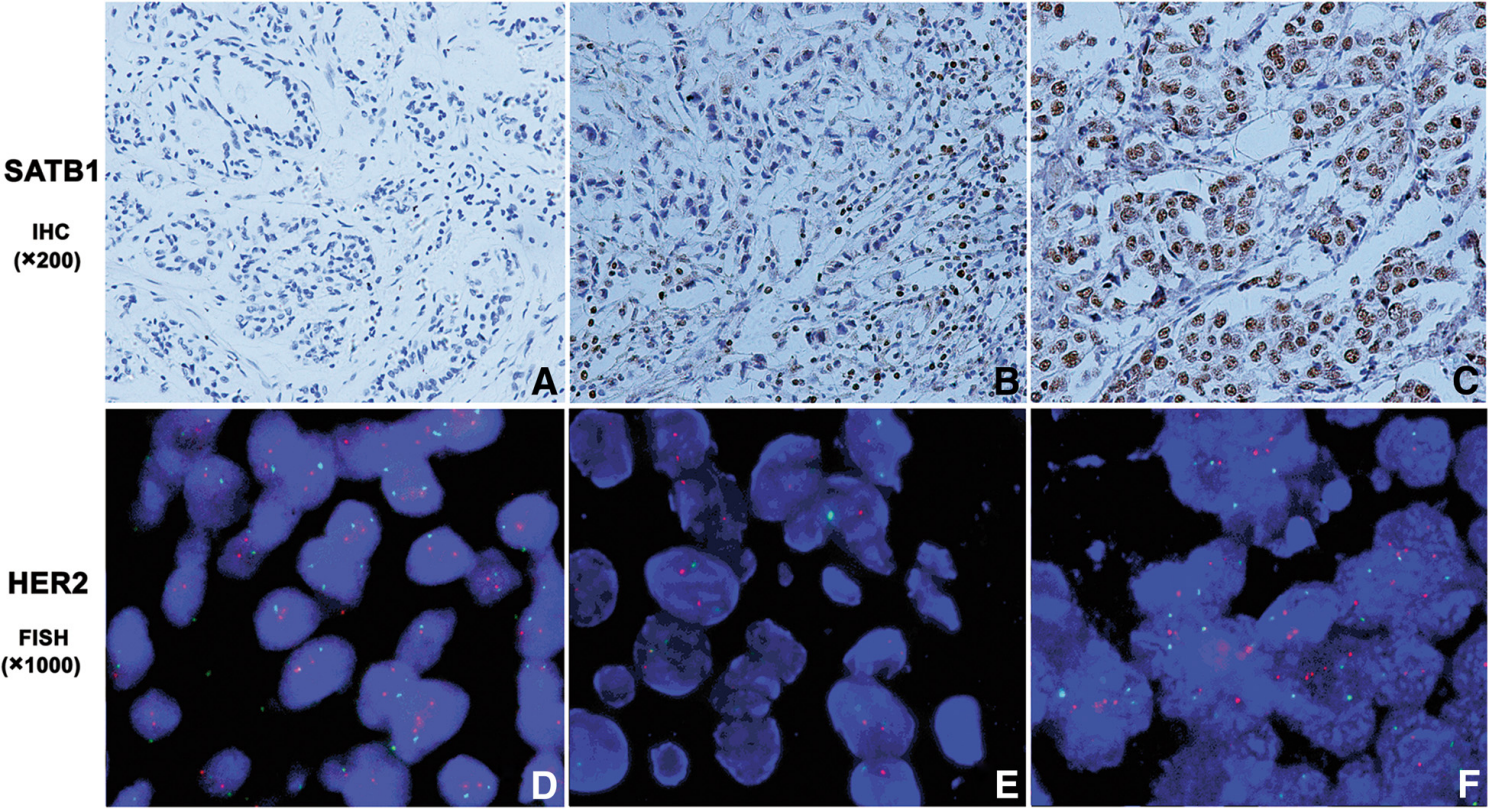
SATB1 expression by IHC(a-c, ×200) and HER2 amplification by FISH(d-e, ×1000). **A** Negative SATB1 expression; **B** SATB1 was positively stained in the infiltrating lymphocytes but negatively stained in breast cancer cells; **C** Positive SATB1 expression; **D** and **E** Non-amplification of HER2; **F** Amplification of HER2.

The staining intensity and the percentage of stained cancer cells were analyzed for SATB1 evaluation. The intensity of the staining was scored as 0 (negative), 1 (weak), 2 (moderate) and 3 (strong). Semi-quantitative grades were determined to assess the percentage of stained cancer cells: 0 (<5% stained cells), 1 (5–25% stained cells), 2 (25–50% stained cells) and 3 (>50% stained cells). The sum of intensity and percentage was calculated, which varied from 0 to 6. Scores of 0–2 were regarded as negative expression, and others with scores 3–6 were defined as positive expression [4]. SATB1 expressed in the infiltrating lymphocytes were not regarded as SATB1 positive for breast cancer cells (Figure 1B).

### HER2 evaluation

The breast cancer Fluorescence in Situ Hybridization Testing Kit (Beijing GP Medical Technologies, Ltd, China) was used to detect amplification of the HER2 gene. Experiments were carried out according to the manufacturer’s recommendations. In brief, the slices were deparaffinized in xylene, digested by protease, fixed in neutral buffered formalin and denatured in the kit solution. Then, hybridization procedure was carried overnight (12–16 h) at 37°C using reagents containing the HER2 DNA probe and the CEP17 DNA probe mixture. After that, the slices were washed in washing buffer and counterstained with 4′, 6-diamidino-2-phenylindole (DAPI) in antifade solution. The slides were stored in dark before signal enumeration. A minimum of 60 tumor cell nuclei were analyzed using a Nikon E-600 fluorescence microscope (Olympus, Japan) equipped with DAPI, FITC and Rhodamine bandpass filters. To rule out false-positive or false-negative results, the positive and negative control tissues were processed together with the cancer tissues in the same staining batch but on different tissue slides.

HER2 was quantified using the ratio of HER2 to CEP17 signal counts according to the American Society of Clinical Oncology (ASCO)/College of American Pathologists (CAP) clinical practice guideline [20,21]. HER2 gene amplification was defined as the HER2/CEP17 signal ratio > 2.0.

### Hormone receptor test

The expression of ER and PR was determined in paraffin-embedded tissue specimens by IHC technique similar to SATB1. Mouse monoclonal anti-human ER and anti-human PR (DKT-3011, MaiXin, China) were used to examine ER and PR protein expression status according to the manufacturer’s instructions.

The expression of ER or PR was designated as positive when more than 1% of tumor cells showed positive nuclear staining per 10 high-power fields, and others were negative expression [22]. ER positive (ER+) and/or PR positive (PR+) were defined as HR positive (HR+), whereas ER and PR simultaneously negative expression (ER-/PR-) was regarded as HR negative (HR-).

### Statistical analyses

Statistical analyses were performed using SPSS 16.0 software (SPSS Inc., Chicago, IL, USA). The two-tailed Chi-squared test or Fisher’s exact test and Spearman’s correlation coefficient were used to evaluate the differences between the expression status of SATB1, HER2, HR and clinicopathologic characteristics in breast cancer patients. Survival curves were calculated using the Kaplan–Meier method and compared by the log-rank test. Univariate and multivariate Cox proportional-hazard analyses were used to explore the effects of SATB1/HER2/HR expression on patients’ survival [4]. *p* <0.05 was regarded as statistically significant.

## Results

### Expression of SATB1, HER2 and HR and their correlations to clinicopathologic parameters in breast cancer tissues

To analyze SATB1 expression status in breast cancer tissues and in corresponding paracancerous normal tissues, SATB1 IHC analysis was performed (Figure 1). SATB1 expression ratio (54.4%, *n =* 92) in cancer samples was significantly higher than that (17.5%, *n =* 7) in corresponding paracancerous normal samples (χ^2^ = 17.701, *p* = 0.000). This result was similar to previous studies [3,17]. Interestingly, our results showed that SATB1 was expressed in the infiltrating lymphocytes within some breast cancer tissues but not in the adjacent non-malignant tissues. To examine HER2 expression status in breast cancer tissues, HER2 FISH analysis was performed (Figure 1). HER2+ expression was observed in 26.0% (*n =* 44) of the breast cancer samples, but HER2 amplification was not found in normal samples. These results were also similar to previous studies [23].

Table 1 showed the relationships between the expression status of SATB1, HER2 and HR and clinicopathologic characteristics in patients with breast cancer, respectively. According to correlation analysis, the expression of SATB1 was associated with higher histological grade in patients with breast cancer (*r =* 0.239, *p* = 0.002). Similar to SATB1, HER2 was statistically associated with higher histological grade (*r =* 0.160, *p* = 0.038). No association existed between HR expression and histological grade (*r = −*0.092, *p* = 0.232). No relevance was observed between the expression of SATB1, HER2 or HR with age, tumor size, tumor type, lymph node status and TNM stage (*p* > 0.05 for each).

### Relevance of SATB1 expression to HER2 amplification and the relationship between their co-expression and breast cancer histological grade

Analysis of the relevance of SATB1 and HER2 showed that 33.7% (*n =* 31) of samples were HER2+ in 92 SATB1+ samples, and that 70.5% (*n =* 31) of samples were SATB1+ in 44 HER2+ samples, but 16.9% (*n =* 13) were HER2+ in 77 SATB1- samples. A significant correlation was found between SATB1 expression and the HER2 status (*r =* 0.191, *p* = 0.013).

**Figure 2:**
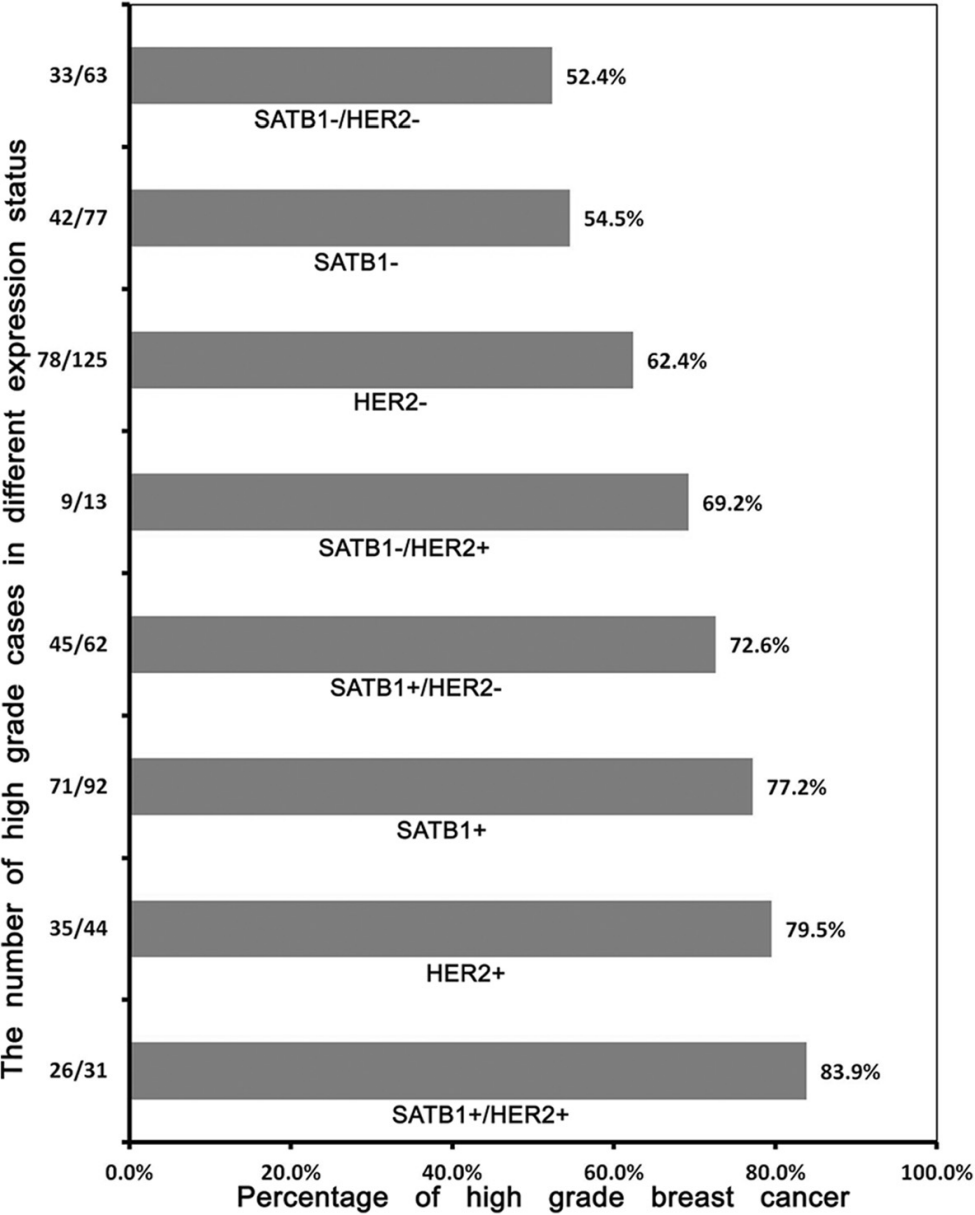
The percentage of high tumor histological grade increased with different expression status of SATB1 and HER2 in breast cancer. The percentage of higher histological grade increased progressively with the status of SATB1 and HER2 expression: SATB1-/HER2-, SATB1-, HER2-, SATB1-/HER2+, SATB1+/HER2-, SATB1+, HER2+, SATB1+/HER2+.

Co-expression of SATB1 and HER2 was correlated with higher histological grade (*r =* 0.306, *p* = 0.003). The percentage of higher histological grade increased progressively with the status of SATB1 and HER2 expression: SATB1-/HER2-, SATB1-, HER2-, SATB1-/HER2+, SATB1+/HER2-, SATB1+, HER2+, SATB1+/HER2+ (Figure 2).

### Association of SATB1/ HER2 expression with HR expression

**Figure 3:**
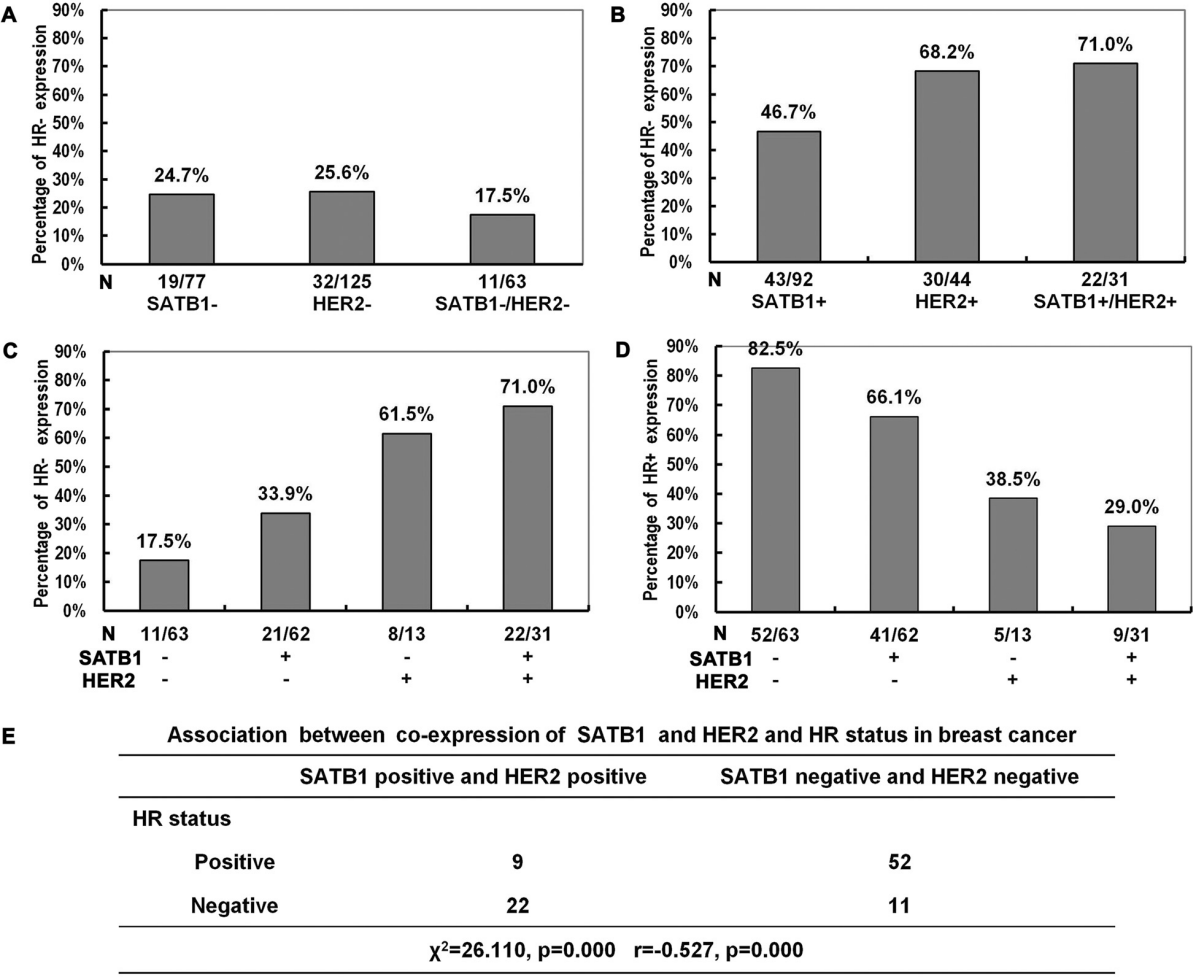
Correlations between the expression status of SATB1/HER2 and HR expression. **A** 24.7% (*n* = 19) samples showed HR- in 77 SATB1- samples, 25.6% (*n* = 32) samples showed HR- in 125 HER2- samples, and 17.5% (*n* = 11) samples were HR- in 63 SATB1-/HER2- samples. **B** HR- expression was found in 46.7% (*n* = 43) of 92 SATB1+ samples, 68.2% (*n* = 30) of 44 HER2+ samples, and 71.0% (*n* = 22) of 31 SATB1+/HER2+ samples, respectively. **C** The effects of SATB1 and HER2 expression status on HR- expression. **D** The effects of SATB1 and HER2 expression status on HR+ expression. **E** SATB1/HER2 co-expression was negatively correlated with HR expression in breast cancer (*r* = −0.527, *p* = 0.000).

Among 169 breast cancer tissues, ER+ and PR+ were observed in 60.4% (*n =* 102) and 53.3% (*n =* 90) samples, respectively, and HR+ was found in 63.3% (*n =* 107) samples. The HR- expression ratio in SATB1-, HER2- and SATB1-/HER2- samples was similar (Figure 3A). On the contrary, the percentage of HR- expression was gradually elevated in SATB1+, HER2+ and SATB1+/HER2+ tissues (Figure 3B). Compared to a large proportion of HR- expression in SATB1/HER2 double positive status, HR+ expression ratio was remarkably lower (Figure 3C and D). In addition, HR- and HR+ sample percentages varied with different SATB1/HER2 status as shown in Figure 3C and D. The results indicated that SATB1 and HER2 co-expression could increase HR- expression ratio.

Similar to previous studies [16], HR- expression was significantly higher in HER2+ samples compared with HER2- samples (χ^2^ = 25.405, *p* = 0.000), and HR- expression was as well increased significantly in SATB1+ samples compared with SATB1- samples (χ^2^ = 8.785, *p* = 0.003). SATB1 and HER2 expression was negatively correlated with HR expression (*r = −*0.228, *p* = 0.003; *r = −*0.338, *p* = 0.000, respectively). Furtherer, a significantly negative correlation was observed between double positive expression of SATB1/HER2 and HR expression (*r = −*0.527, *p* = 0.000) (Figure 3E).

### Analyses of the prognostic value of SATB1, HER2 and HR expression in breast cancer

It was described in our results above that SATB1 or HER2 single positive expression correlated with tumor histological grade. Moreover, SATB1/HER2 co-expression was also significantly associated with higher tumor histological grade (*r =* 0.306, *p* = 0.003). As shown in Figure 2, the percentage of higher histological grade samples increased according to different SATB1 or HER2 status and reached to peak level when SATB1 and HER2 were co-expressed.

**Figure 4:**
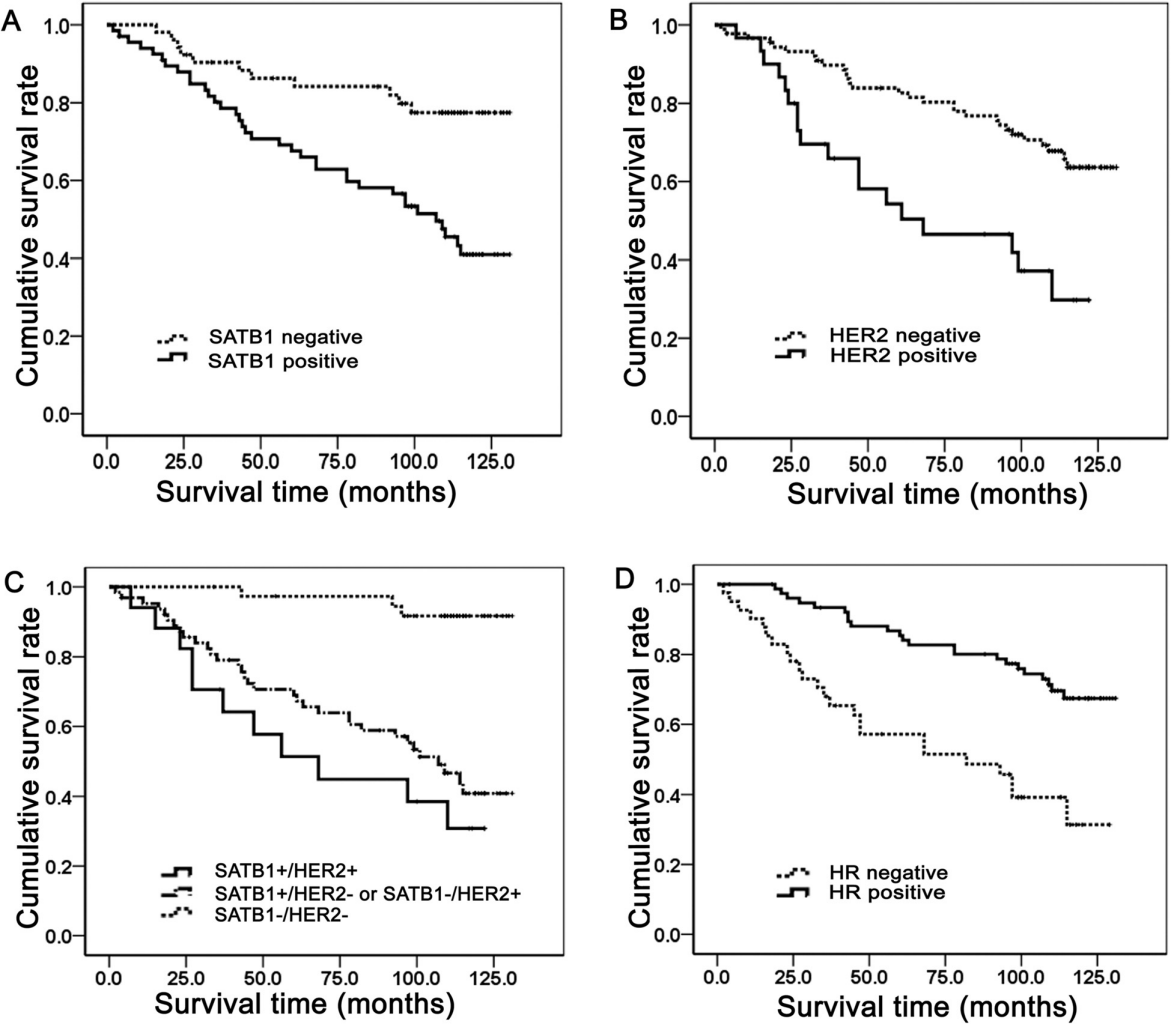
Effects of the expression of SATB1, HER2 and HR to patients’ survival time in breast cancer by Kaplan–Meier analysis. **A** Overall survival for patients with positive SATB1 expression was significantly lower than that with negative expression (SATB1 positive vs SATB1 negative, *p* = 0.001). **B** Overall survival for patients with positive HER2 was significantly lower than that with negative expression (HER2 positive vs HER2 negative, *p* = 0.000). **C** SATB1/HER2 co-expression showed the worst prognosis. **D** Patients’ overall survival with positive HR was significantly higher than that with negative expression (HR positive vs HR negative, *p* = 0.000).

The effect of SATB1 and HER2 expression on breast cancer patients’ survival time was assessed by Kaplan–Meier method. Results showed that patients with SATB1 positive expression had a significantly lower survival rate than those with negative expression (log-rank test, χ^2^ = 11.324, *p* = 0.001) (Figure 4A). Similar result was found in HER2 positive patients (χ^2^ = 12.486, *p* = 0.000) (Figure 4B). Compared to SATB1 or HER2 single positive and SATB1 and HER2 double negative expression, SATB1/HER2 co-expression was prone to have a worse prognosis (Figure 4C). HR expression showed an opposite tendency that HR positive expression inclined to acquire longer survival time (χ^2^ = 16.893, *p* = 0.000) (Figure 4D). Multivariate Cox regression analyses showed that SATB1, HER2 and HR were independent factors for survival rate in breast cancer (Table 2). SATB1 positive patients were liable to have a worse prognosis and the risk exposure was approximately 2.413 times higher (95.0% CI: 1.197-4.846) than that in SATB1 negative patients. Similarly, HER2 positive patients had 2.089 times higher risk (95.0% CI: 1.100-3.969) to a grim prognosis. On the contrary, HR was determined as a protective factor, and its positive expression could reduce the death hazard in breast cancer (95.0% CI: 0.213-0.741).

**Table 2:** **Cox proportional hazards regression analysis for survival**

**Factors**	**Sig. (** ***P*** **value)**	**Relative risk**	**95.0% CI for relative risk**
Age	0.097	1.023	0.996-1.052
Tumor size	0.172	1.789	0.777-4.121
Tumor type	0.594	1.315	0.480-3.604
Histological grade	0.443	1.323	0.647-2.702
Lymph node status	0.352	1.379	0.701-2.712
TNM stage	0.794	1.095	0.554-2.162
SATB1	0.014	2.413	1.197-4.846
HER2	0.024	2.089	1.100-3.969
HR	0.004	0.397	0.213-0.741

## Discussion and conclusions

Human breast cancer is the most malignancy in women and is characterized by multitudinous genetic alterations [24]. SATB1 is found acting as a “genome organizer” that functions as a landing platform to regulate tissue-specific gene expression [25]. Aberrant expression of SATB1 in breast cancer cell lines can make rapid, major changes in gene expression pattern which could alter the cells’ cancerous phenotype [3,26]. Elimination of SATB1 in highly aggressive cancer cell lines alters a large number of gene expression and restrains tumor progress. On the contrary, breast cancer cell lines with ectopic expression of SATB1 experience great changes in their gene expression profile and develop a metastatic phenotype [3]. Our study found that the expression of SATB1 was associated with higher histological grade in patients with breast cancer and SATB1 positive expression had a significantly lower survival rate than those with negative expression. The result was consistent to recent research reported by Heubner, whose work revealed a SATB1 haplotype demonstrating lower activity of SATB1 promotor, and this haplotype associated with improved prognosis [27].

Among expression profile upregulated by SATB1, HER2 is an important regulator to breast cancer progression [3,19]. HER2, a proto-oncogene localized on chromosome 17, is amplified and/or the protein overexpressed in 15-25% of invasive breast cancer [16]. HER2 amplification in breast cancer has been associated with increased invasiveness, tumourigenicity and worse clinical outcomes [28]. What’s more, HER2, as a specific target, could predict the responsiveness to the monoclonal antibody treatment [29]. Thus, HER2 amplification status has become an increasingly important and reliable predictor of patients’ treatment and outcome. In this study, we identified that SATB1 protein expression was associated with HER2 amplification in breast cancer tissues. As has been confirmed, SATB1 could directly upregulate HER2 amplification. Through a series of successive regulation of SATB1 and HER2, the breast cancer performed more malignant activities. However, we also found samples with SATB1-/HER2+ and SATB1+/HER2- expression patterns. This phenomenon reflected that HER2 was not always modulated by SATB1. Some other regulated factors also functioned, such as Chromosome17 polysomy [23,30].

The hormone receptors play an important role in the pathogenesis in breast cancer. By binding with estrogen-responsive elements in the genome, they recruit a series of cofactors that facilitate gene transcription [31]. Consequently, they were regarded as effective target to endocrine therapy. However, recent retrospective studies have suggested that HER2+ tumors may be less sensitive to endocrine treatments [28,32]. In our study, a clear and strong negative association between HER2 and HR in breast cancer was observed. Namely, HR usually negative expressed while HER2 overexpressed in breast cancer, which was in keep with previous studies [10,16]. The inverse association between HER2 and HR levels in clinical specimens is in sympathy with cell line data in a prior study by Pietras [33]. In their study, they introduced HER2 cDNA into MCF7 (which is HER2 low and ER positive cell line) and discovered the transcripts and protein suppression of ER in the transfected cells [33]. This might explain the phenomenon that HER2 amplification/overexpression is consistent with negative HR status. Not only decreasing ER expression, HER2 usually induces the failure of endocrine treatment, such as tamoxifen therapy. In our study, a significantly negative association between SATB1 and HR was also found. Furthermore, the inverse relevance was observed between the co-expression of SATB1/HER2 and HR expression in breast cancer patients. HER2 could regulate ER expression and play a role in the resistance to hormonal, chemo- and radiotherapy [32]. Kobierzycki et al. found a moderate positive correlation between Ki-67 and SATB1 expression and the correlation was even obvious in ER-negative patients (r = 0.291, p = 0.045 independently on the receptor status, and r = 0.392, p = 0.032 in ER-negative tumors) [34], which indicates an indirect role of SATB1 in the cancer cell proliferation. SATB1 could upregulate gene expressions which are associated with tumor cell resistance to apoptosis and multidrug treatment, such as BCL2 and MDR [1,19]. In vitro studies have demonstrated that elevated expression of SATB1 contributes to maintenance of the malignant phenotype and resistance to chemotherapeutic drugs in many other cancers [35,36]. Collectively, SATB1 might inhibit HR expression in cooperate with HER2 and promotes tumor progression or estrogen therapy resistance in breast cancer.

Previous data indicated that HER2 was amplified more commonly in higher histological grade than lower grade in breast cancer [8,16,18]. Similar to these studies, our results showed that HER2 was associated with advanced histological grade in breast cancer. Han et al. [3] reported that SATB1 expression was significantly higher in poorly differentiated tissues. Patani et al. [2] detected SATB1 mRNA increased in high histological grade breast cancer. Accordingly, we also found there was significant correlation between SATB1 and breast cancer histological grade. Moreover, co-expression of SATB1 and HER2 was significantly associated with advanced tumor histological grade in breast cancer patients. Histological grade was an important index of poor differentiation in breast cancer. Our results suggested that HER2+, SATB1+ and SATB1/HER2 co-expression associated with high degree of malignancy in breast cancer. They may play an important role to promote cancer cell proliferation and differentiation. However, no association was observed between HR expression and tumor histological grade. Kaplan–Meier survival analyses demonstrated that SATB1/HER2 single positive and co-expression patients inclined to have poor prognosis, whereas HR positive expression tended to get preferable outcome. Cox regression analyses elucidated that SATB1 and HER2 were independent risk factors for patients’ survival in breast cancer and HR was a protective factor.

Based on the results above, the expression of HER2 had synergistic effect with SATB1 in breast cancer and HR had reverse influence compared to SATB1 and HER2. There might be intrinsic linkage among SATB1, HER2 and HR. Further researches are needed to expound the elaborate mechanism of the crosstalk and regulatory network among SATB1, HER2 and HR.

In conclusion, this study has reported on the relationships between SATB1, HER2, HR expression and clinicopathologic characteristics in breast cancer tissues. SATB1+, HER2+ and SATB1/HER2 co-expression correlated with higher histological grade and were independent risk factors of patients’ survival, which suggested that SATB1/HER2 positive expression weighed highly with poorly differentiated breast cancer and HR were protective factors. Further researches of therapeutics targeting the inner link or crosstalk among SATB1, HER2 and HR would give maximum benefits to patients with breast cancer.
